# Detection of Sensor Faults with or without Disturbance Using Analytical Redundancy Methods: An Application to Orifice Flowmeter

**DOI:** 10.3390/s23146633

**Published:** 2023-07-24

**Authors:** Vemulapalli Sravani, Santhosh Krishnan Venkata

**Affiliations:** 1 Department of Electronics and Communication Engineering, Manipal Institute of Technology Bengaluru, Manipal Academy of Higher Education, Manipal 576104, India; sravani.vemulapalli@manipal.edu; 2 Centre for Excellence in Cyber Physical System, Department of Instrumentation and Control Engineering, Manipal Institute of Technology, Manipal Academy of Higher Education, Manipal 576104, India

**Keywords:** sensor faults, flow measurement, orifice flowmeter, system identification, LPV model, Luenberger observer, neural network, fault detection and isolation

## Abstract

Sensors and transducers play a vital role in the productivity of any industry. A sensor that is frequently used in industries to monitor flow is an orifice flowmeter. In certain instances, faults can occur in the flowmeter, hindering the operation of other dependent systems. Hence, the present study determines the occurrence of faults in the flowmeter with a model-based approach. To do this, the model of the system is developed from the transient data obtained from computational fluid dynamics. This second-order transfer function is further used for the development of linear-parameter-varying observers, which generates the residue for fault detection. With or without disturbance, the suggested method is capable of effectively isolating drift, open-circuit, and short-circuit defects in the orifice flowmeter. The outcomes of the LPV observer are compared with those of a neural network. The open- and short-circuit faults are traced within 1 s, whereas the minimum time duration for the detection of a drift fault is 5.2 s and the maximum time is 20 s for different combinations of threshold and slope.

## 1. Introduction

The current industrial revolution demands automated solutions to existing problems in a user-friendly way. Automated solutions demand many sensors and instruments to achieve the defined challenges. For process monitoring and control, sensors are accurately installed in the necessary locations. The data from the physical sensors must be accurate and updated in shorter spans to capture the finest details of the workstation. Sensor faults may cause false or missing operations, hindering the operation of subsequent dependent systems. The notification and cause of faults for a particular sensor can enable the operator to take the necessary action to reduce the downtime and reduce the monetary loss. Drift faults, step faults (open and short), noise faults, periodic faults, and pulse faults are a few of the different forms of sensor errors that have been documented in the literature [1]. The presence of any of these faults will affect the sensor reading. Hence, the development of sensor Fault Diagnosis (FD) is needed.

Sensors are an integral part of the automised workstation, and they are used for the measurement of level, flow, pressure, temperature, distance, etc. Flow is one of the most widely measured physical variables in industry. There are many different flowmeters in the market, but the choice relies on the user and application. The different flowmeters are mass flowmeters, ultrasonic flowmeters, differential-pressure-based flowmeters, etc. Pressure-based methods are more popular in the market due to their simple construction, cost, and reliability. The orifice flowmeter is a pressure-based flowmeter widely used in industry and can be prone to different faults hindering the operation of the plant [2]. Hence, the present work concentrates on FD in an orifice flowmeter.

For the efficient working and control of any automised process, sensor FD is an absolute necessity and has received attention from many researchers worldwide. The classical method of fault detection is by setting the upper or lower bound; if the parameter of interest is within these limits, then it is a fault-free case. Otherwise, the occurrence of a fault is reported. The choice of the limit is a difficult problem; with a tight limit, disturbances are also treated as faults, and with a larger limit, minor magnitudes of the faults would be ignored. Hence, there is a need for advanced supervision techniques.

Fault diagnosis using principal component analysis in wastewater treatment plants was carried out by Tao et al. [3], and the physical parameters considered for their study were pH, level, dissolved oxygen, and oxidation reduction potential of the wastewater. This study required much historical data for validation. Safizadeh et al. [4] analysed the data from accelerometers and load cells using the K-nearest neighbour classifier and fused the data to indicate vibration faults in bearings. Fault isolation using a Kalman filter was reported in [5] for a flow meter, which was model-based fault diagnosis. An extensive review was carried out for the identification of faults in aircraft fuel systems using machine learning in [6] and for failure detection techniques for air systems in [7].

**Figure 1:**
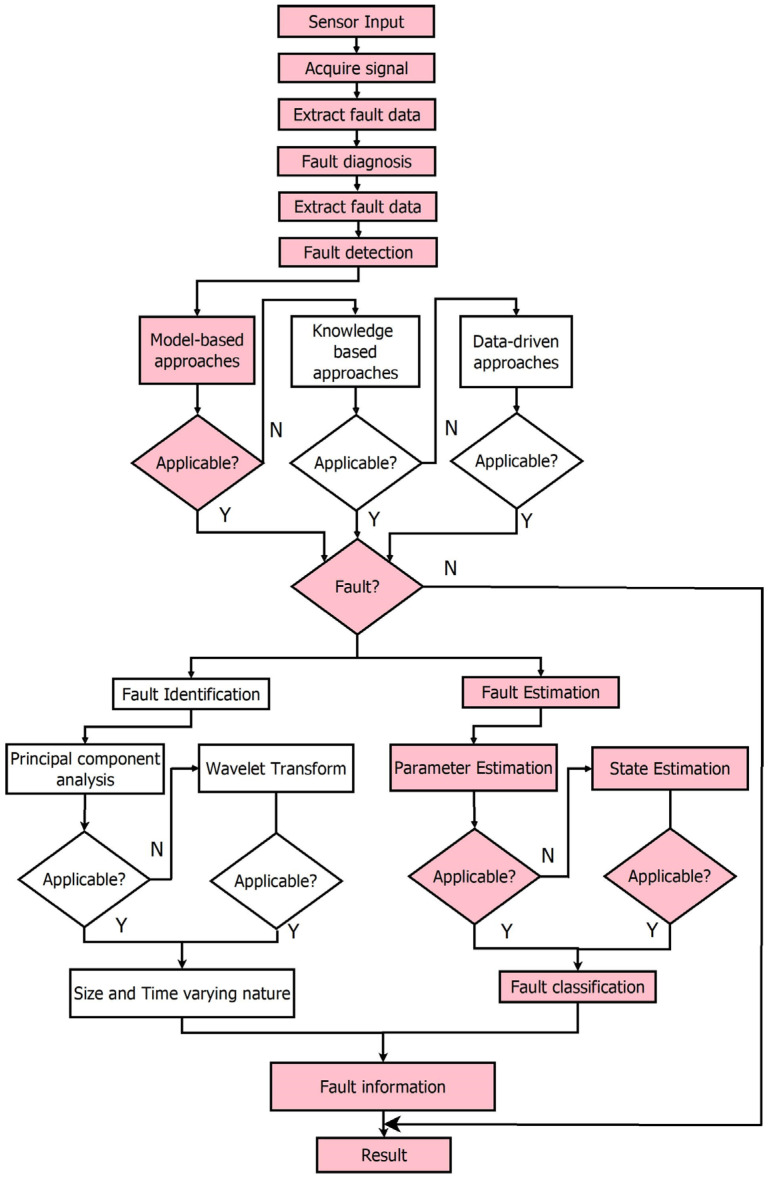
Framework for fault diagnosis.

Li et al. [8] performed a detailed review of different types of sensor faults and diagnosis techniques. Yu et al. [9] reported the use of a radial basis function NN estimator for sensor fault detection in the chemical reactor process. Garramiola et al. [10] proposed a sliding mode observer for the estimation of faults in railway traction drives. A distributed observer based on the algebraic Riccati equation was formulated for sensor FD in multivehicle systems by Qin et al. [11]. An estimation of levels using a chaotic observer was formulated in [12], when the level was disturbed by harsh environmental conditions. Sensor FD was proposed using machine learning algorithms for water reactors by Wang et al. [13]. Furthermore, different types of sensor faults and techniques that were adopted to diagnose them are tabulated in Table 1. The framework to apply fault diagnosis based on a literature survey is given in Figure 1.

**Figure 2:**
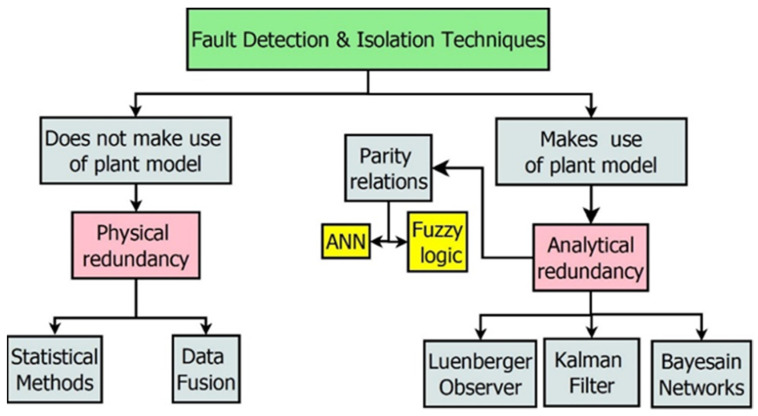
FDI techniques for residual generation.

Fault detection and isolation (FDI) techniques are utilised for the detection of different faults, such as sensor faults, actuator faults, or process component faults. These methods are now crucial for obtaining accurate data from sensors or any other plant component and comprehending the current status of the process components. These techniques depend on residue, which is the difference between the actual value and the current value. These techniques emphasise the creation and evaluation of residues. The residual generation is either based on physical or analytical redundancy. Physical redundancy employs more identical sensors, thereby increasing the working cost. Hence, it is substituted by model-based redundancy techniques (analytical) [19]. The different types of FDI techniques for residual generation are shown in Figure 2. The selection of the technique depends upon the following parameters:

1. Fault detection time;
2. Number of undetected faults;
3. False alarm rate;
4. Ability to isolate faults.

The classification and comparison of different types of FDI techniques are reported in [19,20,21,22,23]. The reported literature splits FDI techniques into model-based [24] or non-model-based techniques [25]. While the non-model-based strategies use the data already there from the sensors or plant for the construction of the FDI technique, the model-based techniques depend on creating a duplicate of the process or a sensor. Model-based techniques have a deterministic approach to handling faults and are thus still practiced compared to recent techniques based on machine learning, which are completely data-driven and stochastic. However, the choice of the scheme rests on the user and application.

The rest of the paper is constructed as follows: Section 2 emphasises model-based FDI. The knowledge gained about the model-based FDI techniques is applied to the detection and isolation of faults in the orifice flowmeter in Section 3. The efficacy of the proposed FDI technique in the form of results is discussed in Section 4. The closing remarks are given in Section 5.

## 2. Model-Based Fault Detection and Isolation

The findings of the experimental station and its fault-free model are equalised by the model-based FDI approaches for the same input; the discrepancy is referred to as a residual signal. Hence, model-based fault detection primarily relies on obtaining a residue signal and evaluating it. The often-used analytical-model-based approaches for residual generation are (i) the observer-based approach, (ii) the parity space approach, and (iii) the parameter-estimation-based approach. Furthermore, the residue is processed and analysed for possible occurrence of the fault.

### 2.1. Observer-Based Approach for Residue Generation

This technique makes use of the residual signal generated by comparing measurements from a process with their estimates generated by an observer (a filter). The observers created for FD are different from those employed for control purposes. State observers are employed for control when it comes to estimating states that the physical sensors are unable to detect. In contrast, FD is dependent on output observers that generate an estimate of the measurements. Observers developed for FD are a special form of filters that generate an estimation of all the states, irrespective of whether they are measured or not.

### 2.2. Luenberger Observer for Residue Generation (LPV System)

The nonlinear system is transposed to a piecewise linear system based on input combinations, and such systems are called LPV systems. Most of the existing systems are nonlinear; hence, for FDI, the observer chosen for such a system in a linear domain is parameter-varying. The LPV models developed are transformed into a state-space model, and the gain matrix, *L*, is calculated. Residue generation for a nonlinear system is not the scope of the present study.

**Figure 3:**
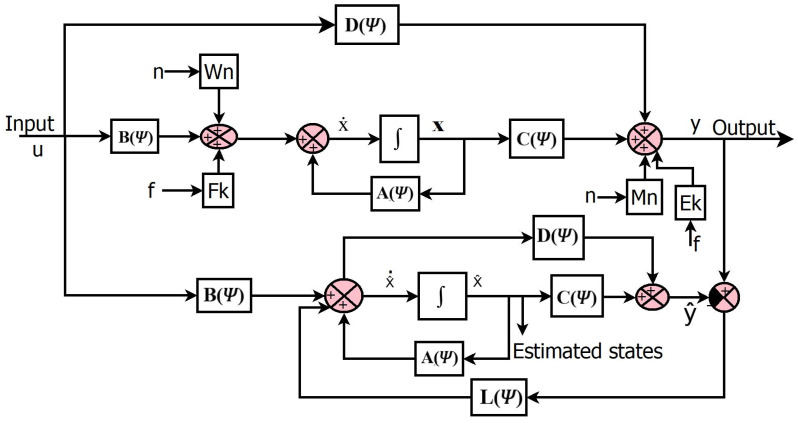
Luenberger observer with fault.

Assume a linear plant with a fault and no disturbance, as shown in Figure 3, with the following equations:

(1) $$\dot{x}=A\left(\Psi \right)x+B\left(\Psi \right)u+F_{k}f+W_{n}n$$

(2) $$y=C\left(\Psi \right)x+D\left(\Psi \right)u+E_{k}f+M_{n}n$$

where Ψ∈P and *P* denotes the parameter set. This representation can describe linear, time-varying (LTV) systems if Ψ=Ψ(t), LPV systems if Ψ∈P, or nonlinear systems if Ψ=Ψ(x). Additionally, *F_k_* and *E_k_* are fault gains, with f as the fault. *W_n_* and *M_n_* are the noise gains, with *n* as noise.

The state equations of the observer are given below:

(3) $$\dot{\hat{x}}=A\left(\Psi \right)\hat{x}+B\left(\Psi \right)u+L\left(\Psi \right)\left(y-\hat{y}\right)$$

(4) $$\hat{y}=C\left(\Psi \right)\hat{x}+D\left(\Psi \right)u$$

(5) $$\dot{x}-\dot{\hat{x}}=\dot{e_{x}}$$

(6) $$\dot{e_{x}}=\left(A\left(\Psi \right)-L\left(\Psi \right)C\left(\Psi \right)\right)e_{x}+n\left(W_{n}-LM_{n}\right)+f\left(F_{k}-LE_{k}\right)$$

(7) $$y-\hat{y}=C(\Psi )e_{x}$$

The choice of *L* shapes the effect of the fault and noise terms on the observer error. The noise and fault models are not considered when calculating the value of *L*. The proposed study calculates the *L* value to reduce the estimation error. Note that if the error system (Equation (6)) is made asymptotically stable by an appropriate choice of observer gain *L*, then a bounded fault and bounded measurement noise *n* will result in the observer error being bounded. The optimal selection of *L* can reduce the fault and noise affecting the system.

After the successful generation of residue, its evaluation is carried out. Residue evaluation contains three stages: residual processing, threshold selection, and decision making. The computation of the root mean square (RMS) is carried out in the residual processing stage, and threshold selection is performed by either the supremum value of the fault-free residual or the user-defined value.

**Figure 4:**
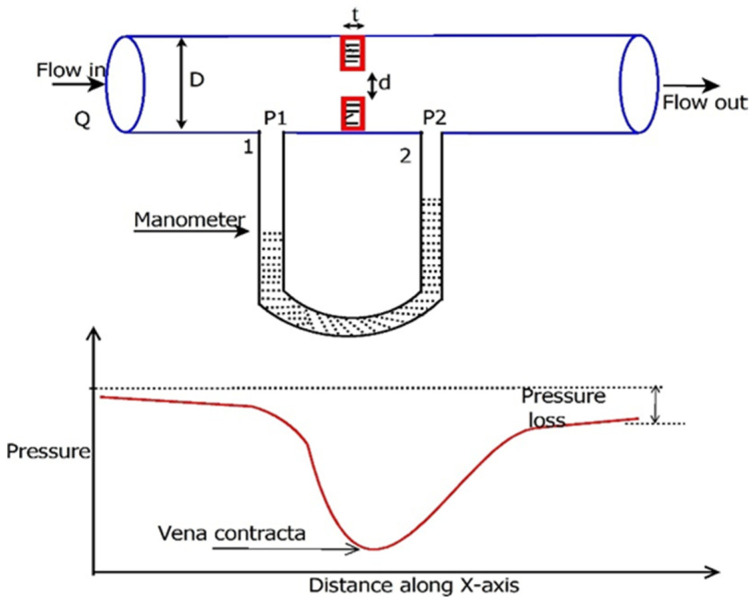
Cross-sectional view of the orifice flowmeter with a manometer with the pressure profile along the x-axis.

In the present study, FDI techniques are applied to analyse the faults experienced by the orifice flowmeter. This flowmeter is widely installed in the field due to its ruggedness and low maintenance [2,26]. An orifice flowmeter with a U-tube manometer is shown in Figure 4. Differential pressure (DP) is measured across the plate at predefined points, and the flow rate can be determined by Equation (8), derived using the concepts of Bernoulli’s theorem and the law of continuity. C_d_ is a quotient of the actual flow rate and theoretical flow rate called the discharge coefficient. Additionally, this measured DP is converted to a 4–20 mA current using a Differential Pressure Transmitter (DPT). The exact measurement of DP plays a crucial role in interpreting the actual flow rate.

The equation for the flow rate is given by

(8) $$Q=\frac{A_{2}C_{d}}{\sqrt{1-\beta^{4}}}\sqrt{\frac{2\left(P_{1}-P_{2}\right)}{\rho }}\;$$

where *P*_1_ and *P*_2_ are pressures at the upstream and downstream tapping of an orifice plate, respectively; ρ is the fluid density; and *β* is the ratio of the square root of total orifice open area *A*_1_ to pipe area *A*_2_, called the beta ratio.

The output of the orifice flowmeter is measured from the DPT, which gives a 4–20 mA output. The open-circuit fault occurs when two terminals of the DPT are disconnected from the measurement loop, resulting in a ‘0’ output. The short-circuit fault is imagined as when two terminals of the DPT are accidentally connected, resulting in an output of less than 4 mA for the entire range of operation. The possibility of a drift fault occurring in the orifice flowmeter is due to leakage in the pressure contacts to the DPT connected across the orifice plate. Considering these scenarios, an attempt is made to detect and identify the type of fault that occurs in the orifice flowmeter. The previous work on orifice flowmeters mainly used a data-driven approach for estimating the discharge coefficient using artificial intelligence [26,27,28,29,30], but fault diagnosis using a model-based approach was not addressed. Hence, this paper focuses on finding three different types of faults that occur in an orifice flowmeter using a model-based approach.

## 3. FDI Techniques Applied to Orifice Flowmeter

**Figure 5:**
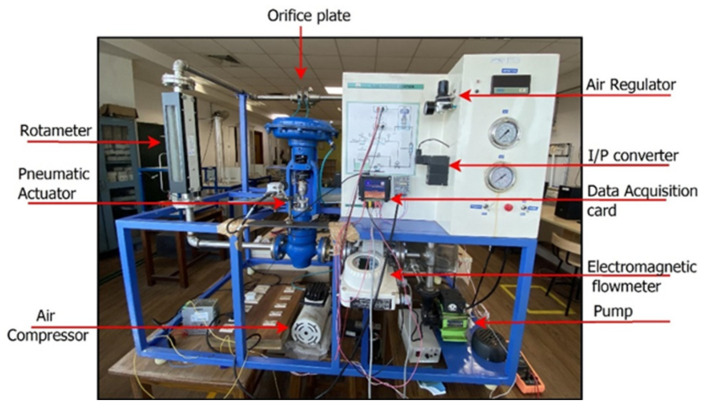
Experimental flow station.

The experimental setup consisting of an orifice flowmeter is illustrated in Figure 5. The quadrant-shaped orifice plate inserted in a ¾-inch pipe has a beta ratio of 0.42 with a thickness of 3 mm. The experimental setup measures the flow rate in the range of 0–1000 LPH. The DPT is connected across the orifice plate at 1D, both upstream and downstream, measuring 4–20 mA. The input current (4–20 mA) gives the signal to the actuator to change its position through the I/P convertor. The variation in the pneumatic actuator’s stem position leads to a change in the flow of fluid to the orifice via a rotameter. The actual flow rate can be visualised in both the electromagnetic flowmeter and rotameter. The presence of the bypass valve bounds the flow rate to 750 LPH in the process.

**Figure 6:**
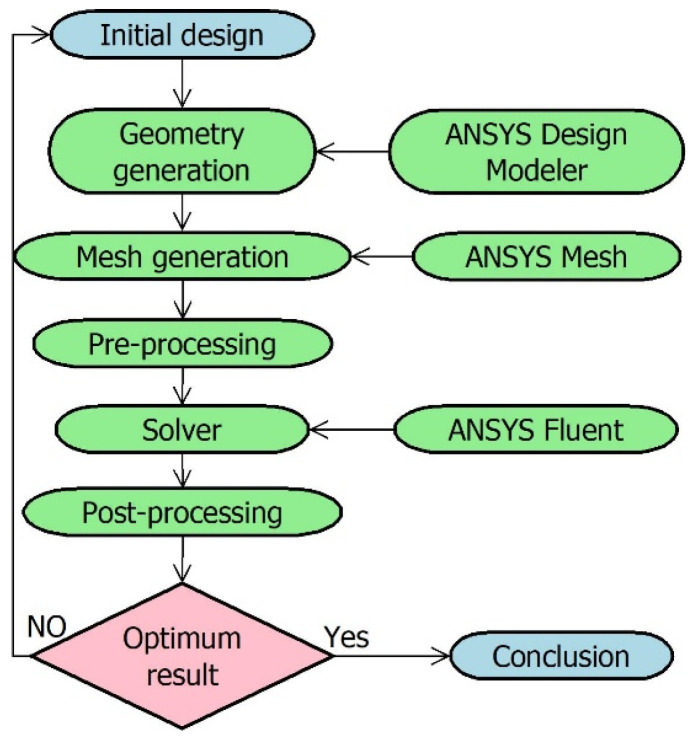
Flowchart for CFD analysis.

**Figure 7:**
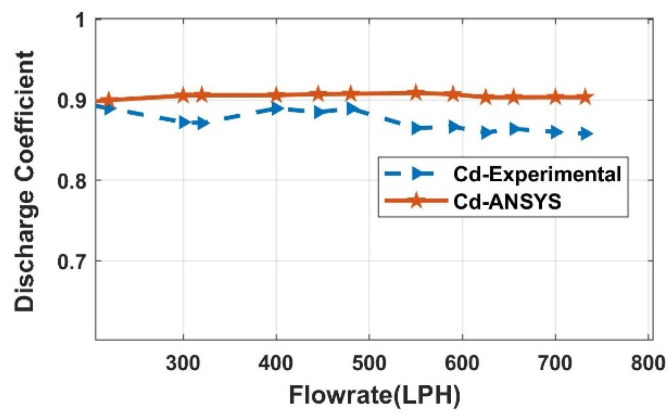
Experimental vs. simulation of C_d_, β = 0.42.

Model-based fault detection techniques are dependent on the model of the physical structure. Hence, the FDI technique requires the model of the experimental orifice flow station. The model is developed after learning the behaviour of the experimental setup of the orifice metre. To further interpret the parametric effect of the beta ratio, density, and velocity on the behaviour of the meter, CFD analysis is considered. CFD helps in building the replica of a real-time process and analysing its behaviour by varying different conditions, which can be difficult real-time. The steps carried out for CFD analysis in ANSYS-19 are shown in Figure 6. The efficacy of the developed model is analysed by comparing its results with experimental results, as shown in Figure 7.

**Figure 8:**
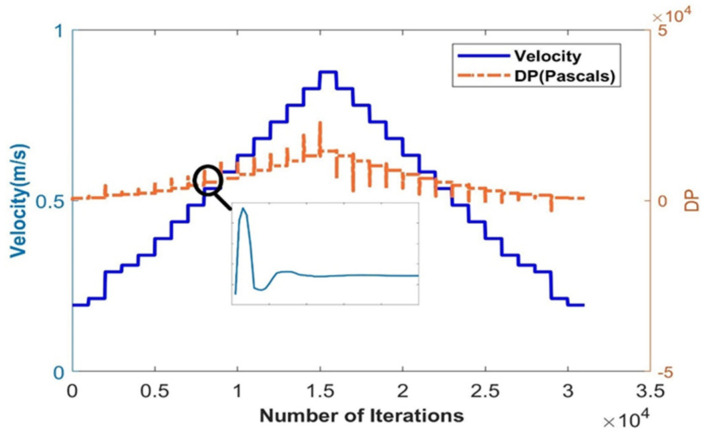
The transient response for a beta ratio of 0.42.

Furthermore, the developed CFD model helps in estimating the DP and therefore the C_d_ value for different orifice plate sizes and fluids. Through the system identification process, transient data recorded from CFD shown in Figure 8 are formulated into a second-order transfer function. Due to the exponential nature of the orifice flow meter, a single transfer function can be used for the entire operation; hence, LPV models are considered.

**Figure 9:**
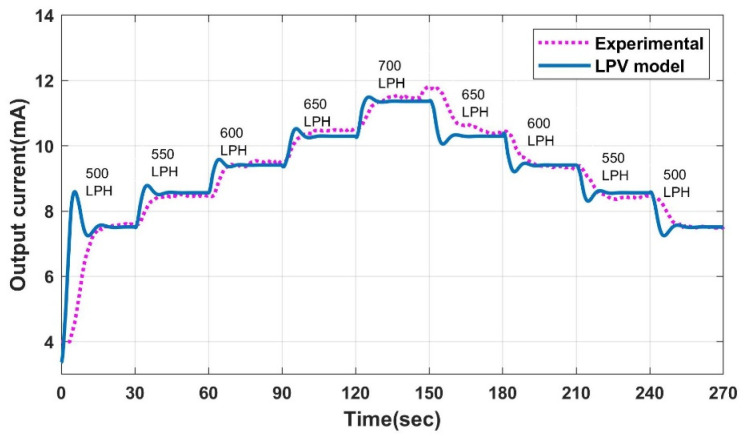
Experimental v/s model output.

The LPV models for the measurement and estimation of flow are formulated and discussed in our previous work [31]. The performance of the LPV models is compared with the experimental system, as shown in Figure 9. The CFD analysis’s inputs are crisp; however, the experimental station includes a delay between the actuator’s movement and the command’s execution. Hence, there is a difference in the initial dynamics of the model and experimental station, but the settling points are the same with less than 5% of error overall.

The state space representation of the LPV models is performed, and using the concept of the Luenberger observer, the gain ‘L’ is calculated. The computation of ‘L’ depends on the selection of the maximum overshoot and settling time, which defines the characteristic equation. The same is developed in [32], which is used for residue generation for fault identification and isolation. The rest of the paper focuses on detecting faults in an orifice flowmeter using the LO-based soft sensor.

**Figure 10:**
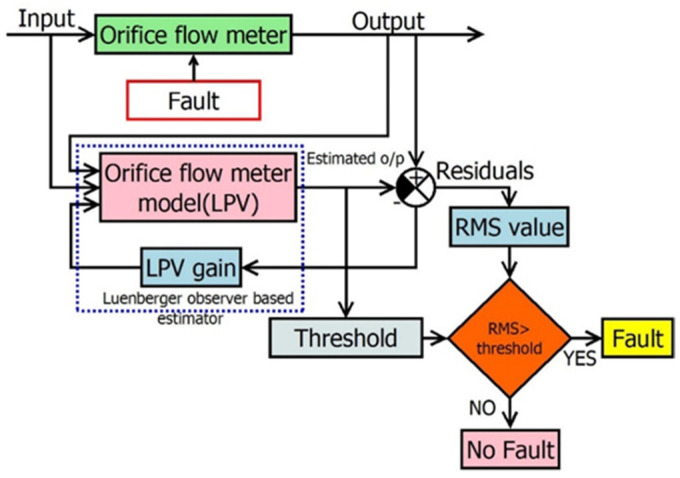
LPV-estimator-based FDI for the orifice flowmeter.

SS based on the LO (Estimator) is designed to detect and isolate the fault in the orifice flowmeter. Due to the exponential nature of the flowmeter response, LPV estimators are designed, where estimators change based on the input velocity, β, and ρ. These estimators help in residue generation, the first part of the FDI technique. Furthermore, the residues are compared with a certain threshold for decision making, as shown in Figure 10. The steps carried out for fault detection and isolation for a given orifice flowmeter are

1. Designing of estimator;
2. Induction of fault;
3. Residue generation: Comparison of estimated value to experimental output;
4. Calculating the Root Mean Square (RMS) value of the residue;
5. Comparison of RMS with the adaptive threshold;
6. Indication of fault;
7. Based on experimental values and fault indications, isolate the different types of faults.

**Figure 11:**
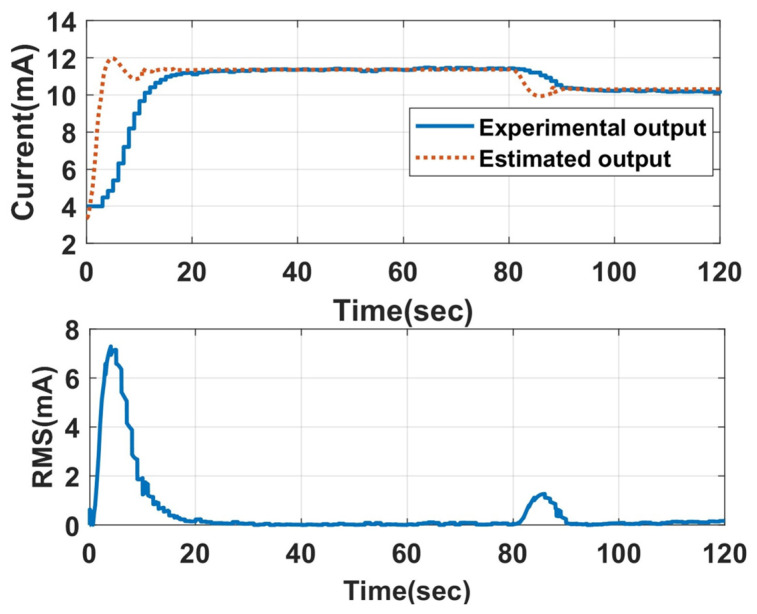
Fault-free output from the LPV estimator.

**Figure 12:**
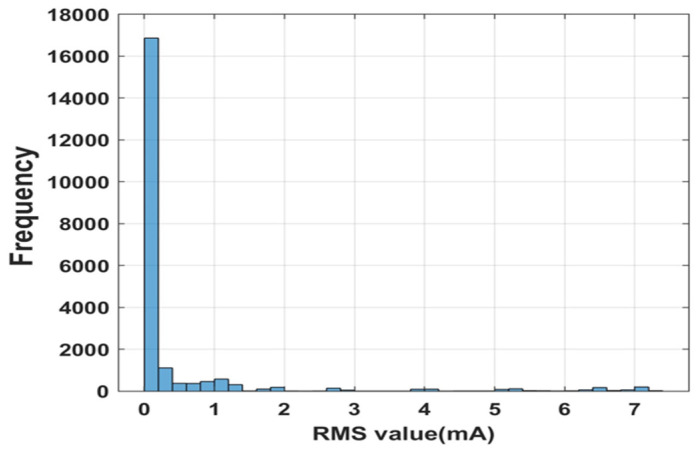
Histogram of RMS values for the fault-free case.

The LO-based LPV estimators are designed considering the required settling time and maximum overshoot. The optimal selection of the L value will aid in filtering the fault impact on the estimated value. Eight of these LPV estimators are built, producing various L values. The gain matrix L, which estimates the output close to the fault-free output, is selected. Fault-free output is the LPV model output, which is compared with the estimator output during the process. The fault-free estimated output from the LPV estimator for 700 LPH and 650 LPH for a beta ratio of 0.42 and water as the working fluid is shown in Figure 11. As observed from Figure 11, the experimental output takes a certain time to reach a steady-state value at the beginning of the cycle, resulting in a high RMS value, which can give a false fault alarm. The histogram of the RMS value of the fault-free case is shown in Figure 12, from which the supremum of the fault-free case is found to be 1.2 mA. This supremum is considered a threshold for fault detection and isolation.

The analysis begins by giving current to the pneumatic actuator to attain 700 LPH for 80 s. The faults were forcefully induced in the output of the flowmeter from the initial 30 s to 60 s for 30 s. First, the drift fault, which is a time-varying offset added to the true value of the sensor, is simulated and then merged with the experimental output.

The equation used for the induction of a drift fault is given as [14]

(9) Y=s∗t−(s∗d)+U

where *Y*, *s*, *t*, *d*, and *U* are the sensor outputs with fault, slope, simulation time, time at which fault is induced, and fault-free sensor value, respectively. The slope values used for the current study are −0.20, 0.20, and −0.10. The time at which the fault is induced is 30 s after running the simulation.

This faulty sensor output is compared with the estimated output, which generates a residue. The RMS value of the residue is calculated and compared with the threshold, which is the supremum value of the fault-free RMS value. If the RMS value is greater than the threshold value, it indicates the presence of the fault in the sensor as ‘1’. The open-circuit and short-circuit faults are induced by making the sensor output ‘0’ and ‘3’ for 30 s, respectively.

The RMS value is made ‘0’ for an initial 15 s at the beginning of the simulation, as it takes the sensor some time to reach a stable value after initialisation. This is performed to avoid false alarms at the beginning of the cycle. Additionally, care is taken so the RMS value turns to zero for 10 s every time the input to the sensor changes, as there is a possibility that the RMS value at that duration may be greater than that threshold, which also results in a false alarm.

**Figure 13:**
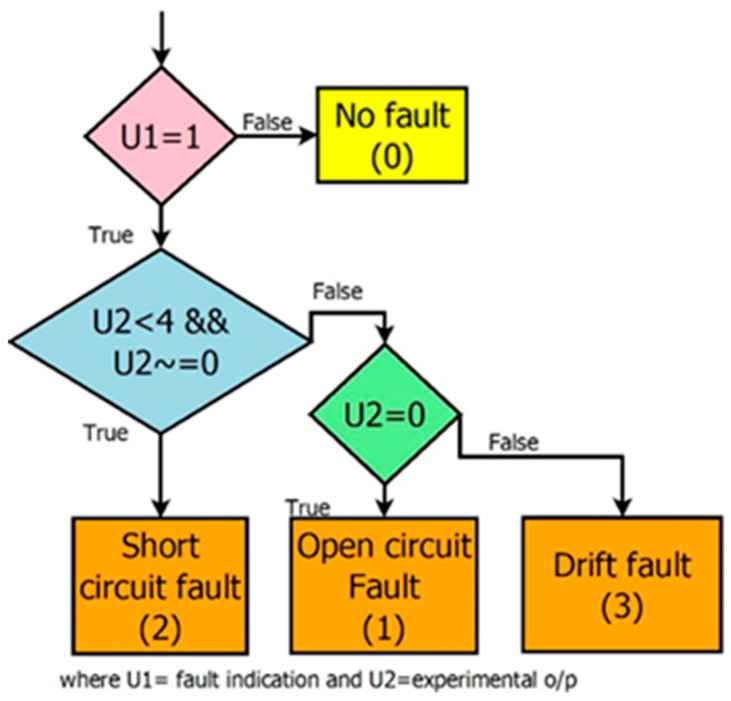
Isolation of three different faults.

After the indication of a fault, it is essential to identify the type of fault. The two features selected for fault isolation are ‘presence of fault’ and ‘experimental output’. Based on these two values, three faults are identified as short circuit, open circuit, and drift faults, as shown in Figure 13. Additionally, the analysis is carried out by setting the threshold as 10% or 15% of the estimated value for a particular input, other than the fixed 1.2 mA, which can result in erroneous fault alarms for small output values. Thus, the supremum value need not be calculated every time the input changes.

### FDI in Orifice Flowmeter Using Neural Network

The residue generation for FDI using the non-model technique is applied to the orifice flowmeter. Neural networks are popularly used for FDI in various applications due to their better accuracy and speed. Based on past input–output data for a specific activity, NNs imitate the operations of the human brain [33,34,35,36]. The structure is developed between the input and output data based on certain mathematical relations. The NN developed in [31] is used for FDI with the beta ratio, density, and Reynolds number as input parameters.

The NN-based FDI technique is applied for the detection of drift faults, open-circuit, and short-circuit faults. The DP estimated from the NN is converted to current based on DP and the current relationship of the orifice flowmeter. The faults are introduced into the sensor, and the effectiveness of the NN is examined for various slope and threshold configurations for the drift fault. Additionally, the response for open-circuit and short-circuit faults is recorded.

## 4. Results and Discussion

**Figure 14:**
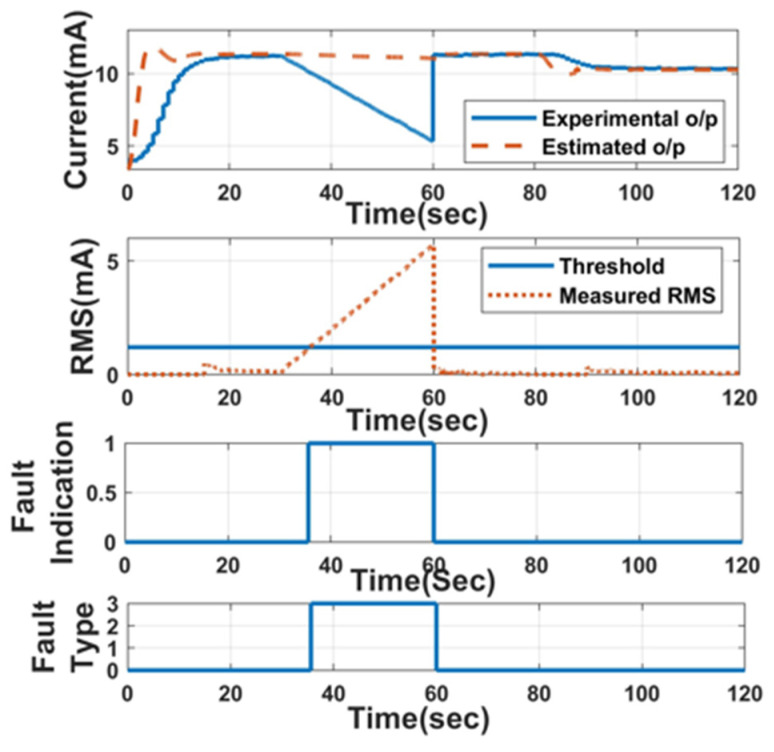
FDI: drift fault: Slope = −20%.

The faults in the orifice flowmeter are forcefully induced to analyse the performance of the LPV estimator for fault detection and isolation. The command is given to the actuator to attain 700 LPH for 80 s and then 650 LPH for the next 80 s. The faults are induced at the 30th second, at the beginning of the cycle for 30 s, and then the experimental output retains its original state. First, a drift fault is induced and identified, as shown in Figure 14, with a threshold of 1.2 mA (supremum value of fault-free RMS). With a higher slope, the proposed FDI technique takes less time for detection after induction compared to the lower slope value.

The response of the proposed FDI technique for the detection of short- and open-circuit faults is also analysed. The drift, short-, and open-circuit faults are distinguished as 3, 2, and 1, respectively. The fault-free condition is labelled ‘0’. For 0.1 s after the fault is eliminated from the initial sensor output, there is the possibility of erroneous fault differentiation as a drift fault in the case of open- and short-circuit faults because the experimental output is attempting to maintain its initial condition. This is due to the condition used for fault isolation. The fault detection times are tabulated in Table 2.

**Figure 15:**
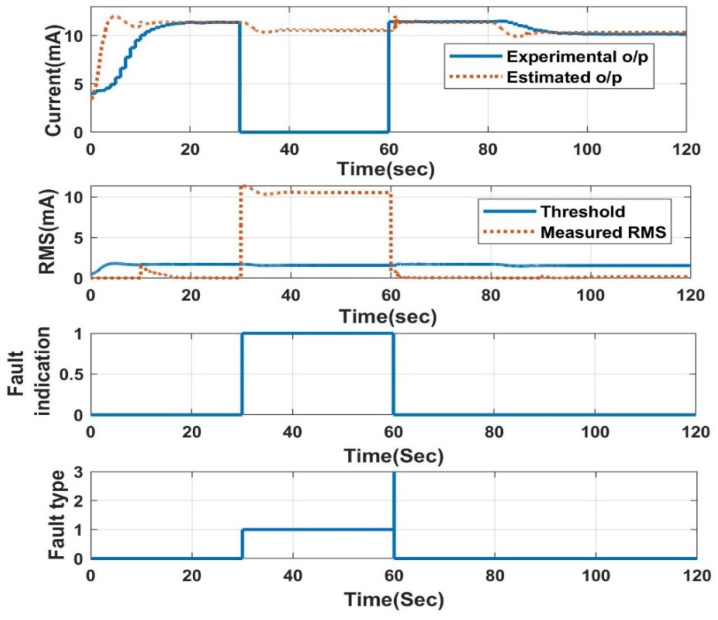
FDI using NN: open-circuit fault.

The response of the LPV estimator with different slopes and threshold values is also analysed. With a higher threshold, the proposed FDI technique takes a long time for detection after induction compared to the lower threshold. Additionally, the induced slope value has a significant impact on the detection time, with a 0.20 slope value, and the fault detection rate is much faster than the 0.10 slope. The response of the NN-based FDI technique for open-circuit fault detection is shown in Figure 15. The comparison between the two proposed FDI techniques regarding the detection time is tabulated in Table 3. Both algorithms perform nearly equally well in terms of detection time, but the NN performs better in a few situations because it does not use the current experimental output for its estimation. The LPV estimator needs to estimate the true value in the presence of faulty experimental output, which causes a certain delay in fault identification.

**Figure 16:**
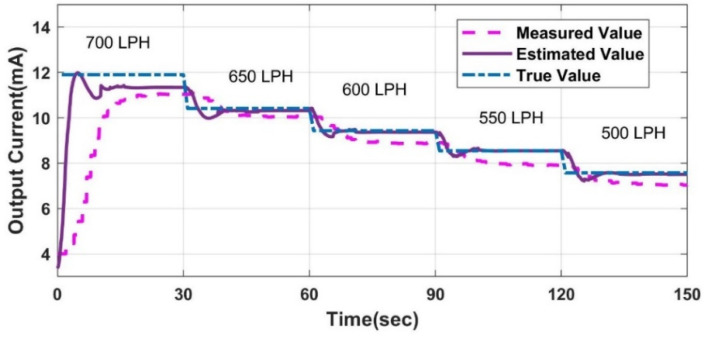
Response of the estimator for the case of disturbance and no fault.

**Figure 17:**
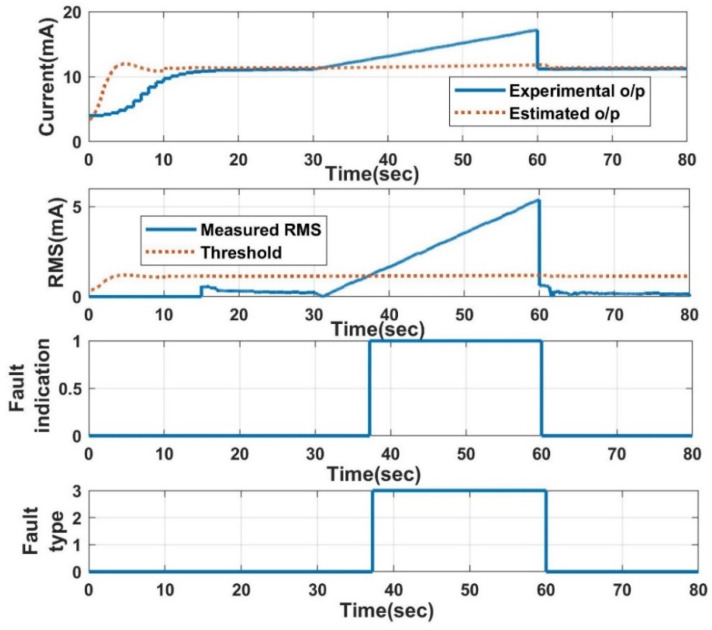
Response of the estimator for disturbance + drift fault (slope 20%).

The performance of the LPV estimator for the combination of disturbance and fault is investigated. The disturbance is induced in the form of a density change by adding 1 kg of sugar to 4 L of water. The estimated value with this disturbance and no fault is visualised in Figure 16. Later, fault + disturbance analysis is implemented by inducing the abovementioned faults. The response of the estimator for drift fault + disturbance is shown in Figure 17. The fault identification time using the LPV estimator for different faults in the presence of disturbances is recorded in Table 4. The technique is also able to track faults efficiently in the presence of the mentioned disturbance.

## 5. Conclusions

Sensor defects in orifice flow meters, such as drift, open circuit, and short circuit, can occur. Therefore, it is necessary to locate and isolate the problems in order to issue an appropriate alarm and take the necessary action. FDI techniques consist of two main components: residue generation and decision making. In the proposed FDI technique, LPV estimators are used for the generation of the residue. LPV estimators are dependent on the mathematical model of the orifice flowmeter. The LPV model is built from the transient data taken from the CFD analysis. The gain value ‘L’ of the LPV estimator is calculated based on different combinations of maximum overshoot and settling time. The optimum L value is selected that can predict the true value in the presence of a fault and help in residue generation.

The RMS values of these residues are compared with a supremum value of fault-free RMS and adaptive threshold (10% or 15% of estimated value) for indication of the fault. Drift faults with different slopes and threshold values are identified. The drift with a higher slope and lower threshold is identified quickly. The threshold of 15% of the estimated value is used to compare with the RMS value for open-circuit and short-circuit faults. The performance of the LPV estimators is equated with the neural network technique used for residue generation in fault identification and isolation. Both techniques showcase almost equivalent detection times. The NN shows better results in a few cases that are independent of the usage of faulty output from the sensor, as it is completely based on fault-free data, while LPV estimators make use of real-time sensor output. The proposed technique is also able to detect faults in the presence of disturbances.

## Figures and Tables

**Table 1:** Previous work in the field of sensor FD.

Author	Type of Sensor Fault	Remarks
Seema Singh et al. [14]	Drift	Applied Neural Network (NN) to transport aircraft for detection of the fault.
R Sun et al. [15]	Step, pulse, noise, periodic, drift	Wavelet energy entropy and support vector regression were used to diagnose faults in a gas turbine control system.
MA A Alobaidy et al. [16]	Stuck, drift	Study of different types of fault detection techniques in the field of robotics.
A Bakdi et al. [17]	Fixed value, gain	Principal component analysis for detection of faults in wind turbines
N Ghosh et al. [18]	Gain, offset, data-loss, out-of-bound	Dempster–Shafer Theory for fault detection in IoT applications.

**Table 2:** Fault identification time of the LPV estimator.

Type of Fault	Threshold	Slope (%)	Time of Fault Detection (s)	Fault Identification Time (s)
Open circuit	1.2	-	30.5	0.5
Short circuit	1.2		30.5	0.5
Drift	1.2	10	43.42	13.42
	1.2	−10	42.58	12.58
	1.2	−20	35.62	5.62
	1.2	20	36.8	6.8

**Table 3:** Comparison of the proposed FDI technique and NN.

Type of Fault	Threshold (%) of the Estimated Value	Slope	Time of Fault Detection (s)		Fault Identification Time (s)	
			LPV Estimator	NN	LPV Estimator	NN
Open circuit	15	-	30.1	30.1	0.1	0.1
Short circuit	15	-	30.1	30.1	0.1	0.1
Drift	15	−0.10	50.155	45.83	20.155	15.8
	10	−0.10	43.76	40.0	13.7	10
	10	−0.20	36.8	35.0	6.8	5
	10	0.20	35.2	36.2	5.2	6.2

**Table 4:** Fault identification time of the LPV estimator with disturbance.

Type of Fault	Threshold (%)	Slope (%)	Time of Fault Detection (s)	Fault Identification Time (s)
Open circuit	15	-	30.5	0.5
Short circuit	15	-	30.5	0.5
Drift	10	10	44.6	13.42
	10	−10	40.01	12.58
	10	−20	34.7	5.62
	10	20	37.2	6.8

## Data Availability

All data are part of paper.
